# Financial debt, worry about debt and mental health in Japan

**DOI:** 10.1186/s12888-023-05235-4

**Published:** 2023-10-17

**Authors:** Andrew Stickley, Aya Shirama, Tomiki Sumiyoshi

**Affiliations:** Department of Preventive Intervention for Psychiatric Disorders, National Institute of Mental Health, National Center of Neurology and Psychiatry, 4-1-1 Ogawahigashi-cho, Kodaira, Tokyo 187-8553 Japan

**Keywords:** Anxiety, Debt, Depression, Japan, Suicidal ideation, Worry

## Abstract

**Background:**

Financial debt has been linked to poorer mental health. However, most research has been undertaken in western countries. This study examined the association between financial debt, worry about debt, and mental health in Japan, where there has been little specific focus on debt and its effects on mental health.

**Methods:**

Data were analyzed from 3717 respondents collected in an online survey in 2023. Information on financial debt and worry about debt was collected with single-item questions. The Patient Health Questionnaire (PHQ-9) and Generalized Anxiety Disorder-7 (GAD-7) scale were used to respectively collect information on depression and anxiety symptoms, while a single-item measure was used to obtain information on a recent history of suicidal ideation. Logistic regression was used to assess associations.

**Results:**

Both financial debt (17.7%) and worry about debt (14.8%) were prevalent in the study sample. In fully adjusted analyses, compared to those with no debt and worry about debt, individuals who were worried about debt but had no debt, or who had debts and were worried about debt had significantly higher odds for suicidal ideation and depressive symptoms. In contrast, having debt but not being worried about debt was not associated with any of the mental health outcomes.

**Conclusion:**

The results of this study suggest that worrying about debt is strongly associated with poorer mental health among Japanese adults. Interventions to address debt and its associated worries may be important for improving public mental health in Japan.

## Introduction

Financial deregulation and increased access to unsecured credit [1, 2] has been accompanied by a growth in personal/household debt in many industrialized nations in recent decades [3, 4]. In 2016 for example, 14% of individuals were unable to make scheduled debt payments in the 28 European Union (EU) countries [5]. Recent research has further shown that between 2002 and 2020 household debt grew steadily in a sample of 26 OECD countries – with falls observed in only three of them [6]. This growth in debt is worrying as although debt can be beneficial in facilitating important life transitions such as acquiring property in young adulthood, and is sometimes essential in the context of economic emergencies, unsecured debt can also have a variety of negative outcomes including delaying marriage, home ownership, the decision to start a family and even retirement [1].

A large body of research suggests that debt may also be associated with worse mental health. Studies among general population samples [7, 8] as well as specific subpopulations such as young [9], middle-aged and older adults [10, 11] have all linked debt to poorer mental health. Further, two systematic reviews have also reported an association between debt and worse mental health [12, 13], while a systematic review and meta-analysis linked personal unsecured debt to mental disorder, depression, and suicide completion or attempt [14]. However, some research has also suggested that the association is nuanced given that it can vary by the type of debt i.e. secured versus unsecured debt [15, 16] or even between specific forms of (unsecured) debt [17]. Indeed, some studies have indicated that other factors might also be important for the debt-mental health association. In particular, stress/worry about debt [18] may play a role in the debt-mental health association, with one study finding that financial concerns were a stronger predictor of mental health than the amount of debt [19]. Similarly, worry about debt was shown to be the strongest predictor of maternal depression in a study among families in the United Kingdom (UK) [20].

Against this backdrop this study will examine the effects of debt and worry about debt on mental health (anxiety and depressive symptoms and suicidal ideation) in the Japanese general population. Like other industrialized nations, in Japan, there has been a growth in access to credit in the past two decades in the context of an expansionary monetary policy and quantitative and qualitative easing policies [21]. This has been accompanied by higher levels of personal debt. For example, in March 2017, the amount of bank card debt (outstanding loans) was approximately 5.6 trillion yen ($50.6 billion) – a growth of 70% over the previous five years [22], while by the end of March 2022 there were 1.16 million people with multiple unpayable loans, with an average combined debt of ¥544,000 ($4469) [23]. Despite this, there has been little systematic focus on debt and its effects in Japan. An earlier study linked debt to late-life depression in adults [24], while more recent research found that student loan debt was associated with psychological distress in graduates (but not current university students) [25]. Importantly, an earlier study found that Japanese suicide completers with unmanageable debt were less likely to engage in help-seeking behavior before their deaths – possibly because of the stigma associated with debt [26].

Thus, this study has three main aims: (i) to examine if debt/worry about debt is associated with mental health outcomes in the Japanese general population. In addition, as there is some evidence that men and women may differ in their forms and levels of debt, as well as in their readiness to seek debt advice [27], and that the strength of the association between debt and mental health can vary between men and women [28, 29], this study will also examine (ii) if the association between debt/worry about debt and mental health differs between men and women. Finally, we will also explore (iii) if differences in the expected length of time it will take to pay off one’s debt is important for the debt-mental health association.

## Methods

### Study participants

Information came from an online survey of the Japanese general population that was undertaken in March 2023. The survey was carried out by Macromill Carenet, a Japanese research company specializing in the healthcare sector. Respondents were part of the company’s online general population web panel. There were three main inclusion criteria for the selection of the study sample: (i) the respondents should be aged 18 and above; (ii) they should be drawn from each of Japan’s 47 prefectures; (iii) the male-female distribution should be representative of the total Japanese population. The final sample consisted of 3717 respondents who were compensated for their participation in the survey. The ethics committee at the National Center of Neurology and Psychiatry, Tokyo, Japan gave permission for the survey (approval number: A2022-096). Informed consent was obtained from all participants.

### Measures

#### Financial debt and worry about debt

Three questions were used to obtain information on the financial debt status of the respondents. First, they were asked, “Do you have any financial debts?” with a yes/no answer option. Those who answered yes were then asked the follow-up question, “How long do you think it will take you to pay off your debts?” with five response options: (i) less than a year; (ii) 1–2 years; (iii) 3–5 years; (iv) 6–10 years; (v) more than 10 years. Finally, all respondents were asked “Are you worried about debt?” Using this information we created a four-category variable that combined information on current debt status with worry about being in debt. Specifically, the first category comprised individuals with no debt and no worry about debt (no debt-no worry), the second category included individuals who were worried about financial debt but did not currently have any debts (worry-no debt), the third category comprised those with current debts but no worry about being in debt (debt-no worry), while the final category included respondents who currently had financial debts and were worried about being in debt (debt-worry).

#### Mental health

Information was obtained on *suicidal ideation* with a single-item question which asked, “Have you ever thought of taking your life, even if you would not really do it?” [30]. Those who answered in the affirmative were then asked to specify if this occurred (i) before the coronavirus pandemic began; (ii) during the coronavirus pandemic; (iii) both before the coronavirus pandemic began and during the coronavirus pandemic. In this study those who stated that they had suicidal ideation during the coronavirus pandemic (options (ii) + (iii)) were categorized as having a recent history of suicidal ideation. *Depressive symptoms* were assessed with the Patient Health Questionnaire (PHQ-9) [31]. This nine-item scale inquires about symptoms of depression (trouble concentrating, tiredness, feeling down etc.,) over the past two weeks. The total scale score ranges from 0 to 27 with higher scores indicating increased depressive symptomatology. Following the suggestion of the scale’s developers in this study those individuals with a score ≥ 10 were categorized as having at least a moderate level of depression [32]. This measure has been previously validated in Japan [33] and had a high degree of reliability in the current study (Cronbach’s alpha was 0.89). Anxiety symptoms were assessed with the Generalized Anxiety Disorder-7 (GAD-7) scale [34]. This seven-item scale inquires about symptoms of anxiety (nervousness, worry, irritability etc.,) over the past two weeks. The scale’s score ranges from 0 to 21 with higher scores indicating increased anxiety. A score of ≥ 10 was used to categorize at least a moderate level of anxiety [34]. Cronbach’s alpha for the scale was 0.92.

#### Covariates

Previous studies were used as a guide when choosing sociodemographic covariates [8, 11, 17]. Information was obtained on the sex (male, female) and age of the respondents. This latter continuous variable was subsequently divided into three age categories: 18–34, 35–59 and ≥ 60. For education respondents were categorized as having either a (i) higher education (two-year college, university, graduate school), or (ii) less than a higher education (junior high school, high school, specialized vocational high school). Three categories were used to assess marital status: (i) married/cohabiting; (ii) never married (single); (iii) divorced/widowed. Information was also collected on household income, which was measured in millions of yen. Initially, three categories were used to assess income level: (i) < 4 million; (ii) 4 < 10 million; (iii) ≥ 10 million (132.93 JPY = 1 USD at the time of the survey). However, as a number of respondents did not provide information for this question (22.9%) and given our wish to keep as many individuals in the analysis as possible, a fourth (iv) ‘missing’ category was subsequently added. Changes in the household financial situation of respondents were assessed as a possible confounder given that income loss might lead to both debt and poor mental health [35]. This was assessed with a question that asked, “How has your household’s economic situation changed during the past year?” Responses were categorized as (i) unchanged; (ii) improved (iii) worsened. Self-rated health was categorized as being either ‘good/very good’, ‘fair’ or ‘poor/very poor’. As previous studies have produced conflicting results on the role of social support in the debt/financial stress-mental health association, showing that it mediates little of the association between debt and mental health [36] but also, that it may moderate the association between financial stress and anxiety in men [37], social support was included in the analysis as a possible mediator of the debt/worry about debt-mental health association. It was measured with a five-item scale that inquired if respondents had anyone who could help them in a crisis, comfort them or listen to them etc. The total scale score could range from 0 to 5 with higher scores indicating more social support. This score was subsequently divided into three categories: (i) high social support (a score of 5); (ii) mid social support (3–4); and (iii) low social support (0–2). As a large number of respondents answered ‘don’t know’ to at least one of these items and would therefore have been excluded from the analysis we also created a ‘missing’ category for this variable. This scale has been previously used to assess social support in the British Household Panel Survey [38, 39] and in a slightly modified form in the Health in Times of Transition (HITT) survey [40] and had a high alpha value in this study (0.94).

### Statistical analysis

Descriptive statistics were first calculated for the study sample stratified by the four-category debt-worry variable. Next, logistic regression was used to assess the association between the debt-worry variable and the mental health outcomes (suicidal ideation, depressive and anxiety symptoms). Separate analyses were run for each mental health outcome using two models. In Model 1 each variable was entered into the analysis separately to examine its unadjusted association with each mental health outcome. Model 2 was a multivariable analysis where all of the variables were included in the analysis at the same time. More specifically, in Model 2 for each mental health outcome the analysis was adjusted for sex, age, education, marital status, household income, household financial change, self-rated health and social support. In addition, when suicidal ideation was the outcome the analysis was also adjusted for anxiety and depressive symptoms, when depression was the outcome the analysis was additionally adjusted for anxiety symptoms, and when anxiety was the outcome the analysis was also adjusted for depressive symptoms. The same analysis was then performed again but this time stratified by sex. Finally, an analysis was undertaken among those with financial debts to examine if differences in the expected time it will take to pay off one’s debts are associated with mental health outcomes. This used the same analytic strategy described above.

All analyses were adjusted for location and performed with SPSS version 24. Results are reported as odds ratios (OR) with accompanying 95% confidence intervals (CI). The level of statistical significance was p < .05.

## Results

The study sample consisted of 3717 individuals, with a mean (SD) age of 52.7 years (18.3) (range 18–89) and slightly more females than males (51.5% > 48.5%). Regarding financial debt, 17.7% (*n* = 659) of the respondents reported that they were currently in debt, while the corresponding figure for being worried about being in debt was 14.8% (*n* = 551). When these variables were combined 79.2% (*n* = 2944) of the respondents had no debt and no worries about debt, 3.1% (*n* = 114) were worried about debt but not currently in debt, 6.0% (*n* = 222) had debts but were not worried about being in debt, while 11.8% (*n* = 437) had debts and were worried about being in debt. Among those who had debts, 21.5% estimated that it would take them 2 years or less to pay off their debts, 24.6% estimated it would take 3–5 years, 15.2% estimated 6–10 years, while 38.7% thought it would take them more than 10 years to pay off their debts. In terms of mental health, a similar percentage of the respondents were categorized as having a recent history of suicidal ideation (9.3%) and anxiety (9.1%), while depression was more prevalent in the study sample (15.6%). The sample characteristics stratified by debt-worry status are presented in Table 1.

**Table 1:** Sample characteristics by financial debt and worry about debt

		Total	No debt – no worry	Worry – no debt	Debt – no worry	Debt – worry
		*n* (%)	*n* (%)	*n* (%)	*n* (%)	*n* (%)
Sex						
Male		1804 (48.5)	1325 (45.0)	63 (55.3)	152 (68.5)	264 (60.4)
Female		1913 (51.5)	1619 (55.0)	51 (44.7)	70 (31.5)	173 (39.6)
Age						
18–34		727 (19.6)	551 (18.7)	36 (31.6)	38 (17.1)	102 (23.3)
35–59		1531 (41.2)	1083 (36.8)	68 (59.6)	120 (54.1)	260 (59.5)
≥ 60		1459 (39.3)	1310 (44.5)	10 (8.8)	64 (28.8)	75 (17.2)
Education						
Higher education		2335 (62.8)	1872 (63.6)	78 (68.4)	134 (60.4)	251 (57.4)
< Higher education		1382 (37.2)	1072 (36.4)	36 (31.6)	88 (39.6)	186 (42.6)
Marital status						
Married/cohabiting		2259 (60.8)	1765 (60.0)	61 (53.5)	160 (72.1)	273 (62.5)
Single (never married)		1026 (27.6)	811 (27.5)	49 (43.0)	40 (18.0)	126 (28.8)
Divorced/widowed		432 (11.6)	368 (12.5)	4 (3.5)	22 (9.9)	38 (8.7)
Household income (yen)						
4- < 10 million		1416 (38.1)	1082 (36.8)	40 (35.1)	96 (43.2)	198 (45.3)
≥ 10 million		272 (7.3)	202 (6.9)	4 (3.5)	35 (15.8)	31 (7.1)
< 4 million		1178 (31.7)	970 (32.9)	36 (31.6)	55 (24.8)	117 (26.8)
Missing		851 (22.9)	690 (23.4)	34 (29.8)	36 (16.2)	91 (20.8)
Household finances						
Unchanged		1906 (52.4)	1629 (56.6)	40 (36.4)	102 (46.4)	135 (31.4)
Improved		269 (7.4)	191 (6.6)	9 (8.2)	36 (16.4)	33 (7.7)
Worsened		1463 (40.2)	1058 (36.8)	61 (55.5)	82 (37.3)	262 (60.9)
Self-rated health						
Good/very good		1662 (44.9)	1325 (45.2)	54 (47.8)	108 (48.6)	175 (40.1)
Fair		1438 (38.9)	1140 (38.9)	39 (34.5)	78 (35.1)	181 (41.5)
Poor/very poor		601 (16.2)	465 (15.9)	20 (17.7)	36 (16.2)	80 (18.3)
Social support						
High		1665 (44.8)	1370 (46.5)	40 (35.1)	92 (41.4)	163 (37.3)
Mid		220 (5.9)	162 (5.5)	6 (5.3)	18 (8.1)	34 (7.8)
Low		432 (11.6)	313 (10.6)	25 (21.9)	24 (10.8)	70 (16.0)
Missing		1400 (37.7)	1099 (37.3)	43 (37.7)	88 (39.6)	170 (38.9)
Suicidal ideation						
No		3062 (90.7)	2476 (92.9)	77 (77.0)	193 (91.5)	316 (79.2)
Yes		313 (9.3)	189 (7.1)	23 (23.0)	18 (8.5)	83 (20.8)
Anxiety symptoms						
No		3379 (90.9)	2716 (92.3)	90 (78.9)	205 (92.3)	368 (84.2)
Yes		338 (9.1)	228 (7.7)	24 (21.1)	17 (7.7)	69 (15.8)
Depression symptoms						
No		3139 (84.4)	2554 (86.8)	74 (64.9)	194 (87.4)	317 (72.5)
Yes		578 (15.6)	390 (13.2)	40 (35.1)	28 (12.6)	120 (27.5)

In unadjusted analyses (Model 1) the same pattern was observed for each of the mental health outcomes (Table 2). Specifically, compared to those with no debt and no worry about debt, being worried about debt but not having debt and having debt and being worried about debt were associated with significantly higher odds for each of the mental health outcomes. In contrast, being in debt but without being worried about debt was not associated with any of the mental health outcomes. When the debt-worry variable was included in the multivariable analysis in Model 2, being worried about debt but having no debt continued to be associated with over two times higher odds for suicidal ideation (OR: 2.18, 95%CI: 1.26–3.78) and depressive symptoms (OR: 2.42, 95%CI: 1.41–4.16). Similarly, in Model 2 being in debt and worried about debt was associated with 2.5 and 1.8 times higher odds for suicidal ideation and depressive symptoms, respectively. None of the debt-worry categories were associated with anxiety in Model 2, while having debt but no worry was not significantly associated with any of the mental health outcomes.

**Table 2:** Financial debt, worry about debt and mental health in the Japanese general population

	Suicidal ideation (*n* = 3317)^†^		Depression (*n* = 3635)^†^		Anxiety (*n* = 3635)^†^	
	**Model 1**	**Model 2**	**Model 1**	**Model 2**	**Model 1**	**Model 2**
	OR (95%CI)	OR (95%CI)	OR (95%CI)	OR (95%CI)	OR (95%CI)	OR (95%CI)
Financial debt						
No debt - no worry	Ref.	Ref.	Ref.	Ref.	Ref.	Ref.
Worry - no debt	4.00 (2.45–6.53)***	2.18 (1.26–3.78)**	3.62 (2.42–5.40)***	2.42 (1.41–4.16)**	3.20 (2.00-5.13)***	1.29 (0.70–2.40)
Debt - no worry	1.20 (0.73-2.00)	1.31 (0.76–2.28)	0.93 (0.62–1.41)	0.87 (0.51–1.47)	0.98 (0.59–1.64)	0.91 (0.48–1.74)
Debt - worry	3.45 (2.60–4.59)***	2.45 (1.76–3.40)***	2.51 (1.98–3.18)***	1.77 (1.28–2.45)***	2.25 (1.68–3.01)***	1.09 (0.73–1.61)
Sex (Female)	1.22 (0.97–1.55)	1.72 (1.30–2.27)***	0.80 (0.67–0.96)*	1.07 (0.84–1.37)	0.89 (0.71–1.11)	1.23 (0.90–1.68)
Age						
18–34	Ref.	Ref.	Ref.	Ref.	Ref.	Ref.
35–59	0.61 (0.47–0.79)***	0.64 (0.46–0.87)**	0.81 (0.65-1.00)	0.77 (0.56–1.05)	0.74 (0.58–0.96)*	0.63 (0.44–0.91)*
≥ 60	0.13 (0.09–0.19)***	0.21 (0.13–0.34)***	0.23 (0.18–0.30)***	0.34 (0.23–0.50)***	0.18 (0.13–0.26)***	0.28 (0.17–0.46)***
Education						
< Higher education	0.92 (0.72–1.18)	0.86 (0.65–1.14)	1.14 (0.95–1.37)	1.01 (0.78–1.29)	1.23 (0.98–1.55)	1.19 (0.87–1.63)
Marital status						
Married/cohabiting	Ref.	Ref.	Ref.	Ref.	Ref.	Ref.
Single (never married)	2.71 (2.12–3.48)***	1.59 (1.15–2.20)**	2.64 (2.18–3.20)***	1.49 (1.11-2.00)**	2.19 (1.73–2.77)***	0.68 (0.47–0.98)*
Divorced/widowed	1.13 (0.74–1.72)	1.67 (1.04–2.68)*	0.95 (0.68–1.31)	1.15 (0.76–1.73)	0.65 (0.41–1.03)	0.59 (0.33–1.05)
Household income (yen)						
4- < 10 million	Ref.	Ref.	Ref.	Ref.	Ref.	Ref.
≥ 10 million	1.02 (0.63–1.65)	1.14 (0.67–1.94)	0.86 (0.58–1.27)	0.69 (0.41–1.16)	1.23 (0.78–1.95)	1.69 (0.94–3.04)
< 4 million	1.05 (0.79–1.40)	0.92 (0.66–1.29)	1.12 (0.90–1.38)	0.88 (0.65–1.19)	1.18 (0.89–1.56)	1.27 (0.86–1.86)
Missing	1.30 (0.96–1.76)	0.92 (0.64–1.30)	1.24 (0.98–1.56)	0.78 (0.56–1.07)	1.53 (1.15–2.04)**	1.42 (0.95–2.13)
Household finances						
Unchanged	Ref.	Ref.	Ref.	Ref.	Ref.	Ref.
Improved	1.90 (1.22–2.95)**	1.16 (0.71–1.89)	1.88 (1.34–2.64)***	1.07 (0.68–1.71)	3.00 (2.02–4.44)***	2.56 (1.52–4.32)***
Worsened	2.19 (1.70–2.82)***	1.51 (1.13-2.00)**	2.04 (1.68–2.47)***	1.27 (0.99–1.63)	2.44 (1.89–3.15)***	1.72 (1.25–2.38)**
Self-rated health						
Good/very good	Ref.	Ref.	Ref.	Ref.	Ref.	Ref.
Fair	1.54 (1.18–2.02)**	1.38 (1.02–1.88)*	2.10 (1.68–2.64)***	1.72 (1.29–2.28)***	1.94 (1.45–2.60)***	1.39 (0.96–2.02)
Poor/very poor	2.51 (1.84–3.41)***	1.43 (0.98–2.09)	5.91 (4.63–7.54)***	4.10 (2.97–5.66)***	5.75 (4.26–7.76)***	2.46 (1.65–3.69)***
Social support						
High	Ref.	Ref.	Ref.	Ref.	Ref.	Ref.
Mid	2.52 (1.59–3.99)***	1.53 (0.91–2.57)	5.02 (3.45–7.32)***	3.22 (2.00-5.19)***	5.93 (3.73–9.41)***	2.81 (1.57–5.04)**
Low	4.05 (2.90–5.64)***	1.71 (1.13–2.57)*	10.96 (8.25–14.56)***	5.24 (3.64–7.53)***	10.03 (7.00-14.36)***	2.81 (1.77–4.47)***
Missing	1.96 (1.47–2.61)***	1.34 (0.97–1.85)	3.80 (2.97–4.87)***	2.63 (1.95–3.54)***	3.95 (2.85–5.49)***	1.83 (1.22–2.75)**
Anxiety symptoms	7.69 (5.83–10.16)***	2.59 (1.79–3.75)***	42.63 (31.70-57.31)***	23.08 (16.64–32.01)***		
Depression symptoms	6.78 (5.26–8.73)***	2.36 (1.67–3.33)***			42.63 (31.70-57.31)***	23.05 (16.59–32.05)***

^†^Individuals included in the multivariable analysis

In the unadjusted Model 1 each variable was entered separately into the analysis. In the multivariable Model 2 all the variables were included in the analysis at the same time

All analyses were adjusted for location

As the final item of the PHQ-9 relates to suicidal ideation, in the suicidal ideation analysis we used the PHQ-8 with a cutoff score ≥ 10 for depressive symptoms

OR: odds ratio; CI: confidence interval; Ref: reference category

*p < .05; **p < .01; ***p < .001

When the analysis was stratified by sex similar results were observed for men and women (Table 3). Specifically, in fully adjusted analyses having debt and worry about debt was associated with over two times higher odds for suicidal ideation in both men (OR: 2.1) and women (2.7), and almost two times higher odds for depressive symptoms in men (OR: 1.8) and women (OR: 1.9). In contrast, having worry about debt but no debt was associated with suicidal ideation in women (OR: 3.4) but not men (OR: 1.6), and with depressive symptoms in men (OR: 2.9) but not women (OR: 1.9). Being in debt but without worry was not associated with mental health in any of the analyses, while none of the debt-worry categories were associated with anxiety in the fully adjusted Model 2.

**Table 3:** Financial debt, worry about debt and mental health in men and women in the Japanese general population

	Suicidal ideation		Depression		Anxiety	
	**Model 1** ^†^	**Model 2** ^††^	**Model 1** ^‡^	**Model 2** ^‡‡^	**Model 1** ^‡^	**Model 2** ^‡‡^
	OR (95%CI)	OR (95%CI)	OR (95%CI)	OR (95%CI)	OR (95%CI)	OR (95%CI)
Men						
Financial debt						
No debt - no worry	Ref.	Ref.	Ref.	Ref.	Ref.	Ref.
Worry - no debt	3.09 (1.48–6.43)**	1.57 (0.71–3.50)	4.09 (2.39-7.00)***	2.88 (1.41–5.86)**	3.25 (1.72–6.12)***	1.15 (0.50–2.66)
Debt - no worry	1.10 (0.55–2.18)	1.05 (0.50–2.20)	0.79 (0.47–1.33)	0.69 (0.35–1.36)	1.06 (0.58–1.94)	1.06 (0.46–2.42)
Debt - worry	3.22 (2.14–4.84)***	2.11 (1.34–3.33)**	2.32 (1.70–3.18)***	1.75 (1.15–2.65)**	1.92 (1.29–2.86)**	0.97 (0.56–1.66)
Women Financial debt						
No debt - no worry	Ref.	Ref.	Ref.	Ref.	Ref.	Ref.
Worry - no debt	5.13 (2.59–10.16)***	3.35 (1.51–7.43)**	2.98 (1.60–5.55)**	1.85 (0.78–4.40)	3.06 (1.49–6.29)**	1.50 (0.58–3.92)
Debt - no worry	1.67 (0.77–3.60)	1.77 (0.76–4.14)	1.14 (0.57–2.28)	1.14 (0.48–2.71)	0.74 (0.26–2.06)	0.52 (0.16–1.72)
Debt - worry	4.12 (2.73–6.22)***	2.71 (1.65–4.43)***	2.67 (1.84–3.86)***	1.91 (1.12–3.26)*	2.75 (1.78–4.24)***	1.36 (0.75–2.49)

^†^1649 men, 1726 women included in the analysis; ^††^1628 men, 1689 women included in the analysis; ^‡^1804 men, 1913 women included in the analysis; ^‡‡^1772 men, 1863 women included in the analysis

In the unadjusted Model 1 only the debt variable was entered into the analysis. The multivariable Model 2 was additionally adjusted for age, education level, marital status, household income, household financial change, self-rated health, and social support. In addition, the suicidal ideation analysis was also adjusted for depression and anxiety, while the depression analysis was adjusted for anxiety and vice versa

All analyses were adjusted for location

As the final item of the PHQ-9 relates to suicidal ideation, in the suicidal ideation analysis we used the PHQ-8 with a cutoff score ≥ 10 for depressive symptoms

OR: odds ratio; CI: Confidence interval; Ref: Reference category

*p < .05; **p < .01; ***p < .001

When the analysis was restricted to those with debts, compared to those who expected to pay off their debts in two years or less, adults who expected it would take 3–5 years (OR: 2.88, 95%CI: 1.31–6.35) and more than 10 years (OR: 2.18, 95%CI: 1.01–4.71) had over two times higher odds for a recent history of suicidal ideation (Table 4). There was no association between expected debt duration and depressive symptoms, while the only association with anxiety was for individuals expecting to take 6–10 years to pay off their debts who had significantly reduced odds for anxiety (OR: 0.29, 95%CI: 0.09–0.91).

**Table 4:** Estimated time to pay off financial debts and mental health in the Japanese general population

	Suicidal ideation		Depression		Anxiety	
	**Model 1** ^†^	**Model 2** ^††^	**Model 1** ^‡^	**Model 2** ^‡‡^	**Model 1** ^‡^	**Model 2** ^‡‡^
	OR (95%CI)	OR (95%CI)	OR (95%CI)	OR (95%CI)	OR (95%CI)	OR (95%CI)
Time to pay off debt						
≤ 2 years	Ref.	Ref.	Ref.	Ref.	Ref.	Ref.
3–5 years	2.14 (1.08–4.22)*	2.88 (1.31–6.35)**	1.32 (0.76–2.30)	1.83 (0.85–3.93)	0.93 (0.48–1.80)	0.69 (0.28–1.72)
6–10 years	1.43 (0.66–3.11)	2.15 (0.85–5.44)	0.99 (0.52–1.90)	2.10 (0.89–4.96)	0.50 (0.21–1.18)	0.29 (0.09–0.91)*
> 10 years	1.33 (0.69–2.54)	2.18 (1.01–4.71)*	1.26 (0.76–2.11)	1.96 (0.96-4.00)	0.97 (0.54–1.77)	0.70 (0.30–1.64)

^†^ 610 individuals included in the analysis; ^††^601 individuals included in the analysis; ^‡^659 individuals included in the analysis; ^‡‡^649 individuals included in the analysis

In the unadjusted Model 1 only the time period variable was entered into the analysis. The multivariable Model 2 was additionally adjusted for sex, age, education level, marital status, household income, household financial change, self-rated health, and social support. In addition, the suicidal ideation analysis was also adjusted for depression and anxiety, while the depression analysis was adjusted for anxiety and vice versa

All analyses were adjusted for location

As the final item of the PHQ-9 relates to suicidal ideation, in the suicidal ideation analysis we used the PHQ-8 with a cutoff score ≥ 10 for depressive symptoms

OR: odds ratio; CI: Confidence interval; Ref: Reference category

*p < .05; **p < .01

## Discussion

This study examined the association between debt, worry about debt and mental health in a sample of 3717 Japanese general population adults drawn from an online survey. Both debt (17.7%) and being worried about being in debt (14.8%) were prevalent in the study sample. There was some indication that worry about being in debt rather than actually being in debt might be more important for mental health. Specifically, compared to those with no debt and no worry, in fully adjusted analyses individuals who were worried about being in debt but did not have debts, and those who had debts and were worried about being in debt had significantly higher odds for suicidal ideation and depressive symptoms. In contrast, being in debt without worry was not associated with any of the mental health outcomes. Further analyses showed that the association between debt/worry about debt and mental health was similar in men and women. Finally, individuals who thought it would take them a longer amount of time to pay off their debts had significantly higher odds for suicidal ideation but not depressive or anxiety symptoms.

Previous research has indicated that there is a strong association between personal/household debt and poorer mental health [12–14]. In particular, debt is regarded as a stressor [41] that may affect mental health either directly or indirectly through its negative effect on e.g. social relationships or coping capacity [36]. However, the results from this study are more in line with those that have linked worry about debt [20], stress/worry about debt [18] and financial concerns [19] to poorer mental health. It is uncertain why worry about debt was seemingly more strongly associated with worse mental health than actual debt itself, although the finding that debt without worry was not associated with any of the mental health outcomes does provide support for the notion that not all debt is necessarily detrimental for mental health and that debt may have a beneficial effect in some instances [42].

More generally, the finding that worry about debt was linked to worse mental health is in line with research that has linked worry (i.e., “a chain of thoughts and images, negatively affect-laden and relatively uncontrollable” [43]) with poorer mental health in clinical, subclinical and nonclinical populations [44], and with the results of a recent study linking worry with suicidal ideation in Japanese adolescents [45]. Interestingly, although excessive worry is a diagnostic criterion of generalized anxiety disorder [46], we found that worry about debt was associated with suicidal ideation and depressive symptoms but not anxiety symptoms in analyses adjusted for co-occurring mental health conditions. It has been suggested that chronic worriers may experience hopelessness and then depression when they realize that they cannot avoid future uncontrollable events [47], while a more recent study reported that it was the uncontrollability of one’s thoughts that might be the transdiagnostic factor linking worry and outcomes such as depression and suicidal ideation [48].

When the analysis was restricted to those individuals who had debts, a longer estimated time period to pay off one’s debts was associated with higher odds for suicidal ideation but not depression or anxiety symptoms. Before discussing this finding it is important to note that caution should be exercised when interpreting this result given the smaller number of individuals included in the analysis and the possibility that the observed associations might simply have been an artifact of the way the debt-time variable was operationalized. To the best of our knowledge, as yet, there has been no previous research on the effects of the estimated length of the debt repayment period – although several studies have focused on the effects of long-term debt on mental health. This research has produced conflicting findings with some studies showing an association between longer-term debt and poorer mental health [16, 41], while others have not [49]. Thus, we can only speculate how the perceived length of the probable debt repayment period might affect mental health. For example, it might be associated with the perceived manageability of the debt – with a longer time period making the debt either easier or more difficult to service. This might be relevant as a recent study from the UK linked greater perceived difficulty in managing debt to worse mental health [50].

The findings of this study should be considered in the context of a number of limitations. First, we had no information on the specific form of the debt or the total number of debts. This is an important limitation as previous research has indicated that there might be differences in the association between secured (e.g. a mortgage) and unsecured debt (e.g. a credit card loan) in terms of mental health outcomes [15, 16], while a higher number of debts has also been linked to a greater prevalence of poorer mental health [8]. Relatedly, we also had no information on when the indebtedness began, its ongoing length or whether it occurred specifically in the context of the COVID-19 pandemic, where increased household debt has been linked to poorer mental health [51]. Second, this study was cross-sectional and so it was not possible to determine the direction of the observed associations. In an earlier longitudinal study an initial association between worry about debt and maternal depression was accounted for when controlling for baseline depression [20], while more recent research has indicated that common mental disorders are associated with difficulty in subsequently paying off debts [52]. Third, it has been suggested that poorer mental health might result in higher levels of worry about debt, while global measures of worry about debt might simply reflect other concerns about such things as future income levels or the possibility of unemployment [12]. Fourth, it should also be noted that because information was collected on suicidal ideation in relation to the coronavirus pandemic (i.e. over a period of two years), it was not possible to ascertain if these suicidal thoughts were still current or might have already passed at the time of the survey, or if indeed such thoughts might have even preceded being in debt. Fifth, a small number of respondents (3.1%) reported that they were worried about debt but did not have debts. While we cannot discount the possibility that these individuals might have had debts and failed to report them (either deliberately or mistakenly) other research has highlighted that many people are afraid of/worry about taking on debt [53] and that anticipated debt may be associated with detrimental outcomes [54]. Finally, as our study sample was obtained online, it is possible that it might not be fully representative of the underlying population in regard to such factors as its education and income levels.

## Conclusion

This study showed that debt and worry about debt were prevalent in the Japanese general population and that both being in debt and worrying about that debt and worrying about debt in the absence of debt were strongly associated with poorer mental health in Japanese adults. Together with the results from earlier studies this finding provides support for the idea that both subjective and objective aspects of debt may be important for mental health. Given the stigma, embarrassment and shame that can be associated with debt [55] and that it may be linked to a reluctance to seek help in Japanese individuals with mental health problems [26], the results of this study suggest that efforts to increase awareness of debt, its effects, and effective responses both among the general public and among mental health [56] and other key health professionals may be important for improving public mental health in Japan.

## Data Availability

The datasets generated and/or analyzed during the current study are not publicly available due to the terms of the data provision contract but are available from the corresponding author upon reasonable request.
